# Setae from Larvae of the Northern Processionary Moth (*Thaumetopoea pinivora, TP)* Stimulate Proliferation of Human Blood Lymphocytes *In Vitro*

**DOI:** 10.1371/journal.pone.0113977

**Published:** 2014-12-22

**Authors:** Göran Holm, Margareta Andersson, Monica Ekberg, Bengt Fagrell, Jan Sjöberg, Matteo Bottai, Magnus Björkholm

**Affiliations:** 1 Department of Medicine, Division of Hematology, Center for Molecular Medicine, Karolinska University Hospital Solna and Karolinska Institutet, Stockholm, Sweden; 2 Division of Biostatistics, Institute of Enviromental Medicine, Karolinska Institutet, Stockholm, Sweden; University of San Francisco, United States of America

## Abstract

Larvae of the Northern pine processionary moth (*Thaumetopoea pinivora, TP)* carry microscopic needles (setae), which by penetrating skin and mucous membranes, may cause inflammatory/immune derived symptoms in man. In the present study the stimulatory effects of setae on human blood lymphocytes *in vitro* was investigated. Blood mononuclear cells were separated from venous blood or buffy coat of ten healthy individuals, six previously exposed to setae and four with no known exposure. Lymphoproliferation was measured as uptake of ^3^H-thymidine. Setae were prepared from TP larvae. Setae and saline setae extracts stimulated proliferation of T-lymphocytes in the presence of monocytic cells. Stimulation was pronounced in cells from persons who had been exposed to setae, and weak in cells from non-exposed donors. Chitin also induced lymphocyte proliferation in most donors, but to a lesser extent and independently of donor's previous exposure to setae. In conclusion, setae contain molecules that in the presence of monocytes activate human T-lymphocytes to proliferation. The antigenic nature of stimulatory molecules was supported by the significantly stronger lymphocyte response in persons previously exposed to setae than in non-exposed donors. The nature of such molecules remains to be defined.

## Introduction

Processionary moths with larvae that carry urticating hairs (setae) are present worldwide. The Northern processionary moth (*Thaumetopoea pinivora*, TP) is mainly localised around the southern coasts of the Baltic Sea [1].

TP larvae carry setae of microscopic size (100–500 μm long and 3–7 μm in diameter), that can be released and transferred to humans and animals via air or by direct contact [2]. Setae are composed of chitin, a major component of the exoskeleton of insects, together with proteins and other constituents. Setae have sharp tips that facilitate the penetration into skin or mucous membranes, thereby provoking local or general symptoms. The skin sites around setae may show itching with swelling, maculopapular redness and blister formation [3]–[5]. Inhaled setae may cause asthmatic reactions and nasal or oral contacts may give rhinitis and local inflammation. Severe gastrointestinal distress has been noted after ingestion [1].

A population present at the southern part of the island Gotland in the Baltic sea has increased in size and become a major concern among people living in or visiting the area [2]. In a poll among persons who stayed in the area during the summer 2005, 20% reported symptoms after contacts with larvae, one third of them described severe symptoms [6].

The mechanisms behind symptoms and diseases induced by setae components are only partly known. Acute allergic reactions towards defined setae associated proteins have been described, mainly in heavily exposed persons [5]. On rare occasions, general and life-threatening anaphylactic reactions may develop [7]. However, previously non-exposed persons contracted symptoms 6–12 hours after experimental skin exposure [3]. These observations suggest that mechanisms other than rapid IgE-mediated allergic reactions contribute to the symptoms. The role of proteins, chitin, lipoproteins and other setae constituents in host reactions following seta exposure needs to be investigated.

The aim of this study was to elucidate lymphocyte-mediated immune reactions triggered by setae and setae proteins from TP larvae. The hypothesis that setae activate cell-mediated immune reactions was tested by *in vitro* cultivation of human blood mononuclear cells in the presence of setae or setae extracts from TP. In addition the role of chitin was evaluated.

## Material and Methods

### Study population

Four healthy persons, two women and two men, aged 25–70 years, with no known previous exposure to setae and six persons, two women and four men, aged 25–80 years who had been living in setae contaminated areas, acted as donors. Buffy coats were received from the Department of Clinical Immunology and Transfusion Medicine, Karolinska University Hospital Solna, Stockholm. The identity of buffy coat blood donors was coded and unknown to us. The study was approved by the Regional Ethical Review Board in Stockholm, Sweden

### Sampling of TP caterpillars and preparation of setae

In accordance with the Swedish Right of Public Access, larvae were collected from pine trees on southern Gotland during late July and early August when larvae had reached their final maturation before they move down from the pine trees to pupate. They were stored at −80°C. Setae were collected from setae mirrors of partly thawed larvae by picking them with a forceps. The setae were washed, concentrated to 400,000/ml. It was assured that the larval integument was not damaged to avoid contamination by components, which may be toxic to lymphoid cells. After three washes in phosphate buffered saline (PBS) pH 7.4, containing 2% human serum albumin with penicillin (100 U/ml) and streptomycin (100 µg/ml), setae were stored in PBS at −80°C.

### Setae extracts

Setae suspended in PBS were sonicated in a Bandelin Ultrasonic Homogeniser – Sonopuls (Gallay, Melbourne, Australia) at 18 W for 6 min. Remaining particles were removed by centrifugation at 3200 g. The setae free supernatants were stored at −80°C. The protein content in supernatants was measured by the BCA protein assay (Bio-Rad Laboratories, Solna, Sweden) and read at 620 nm on an ELISA reader Thermo Multiscan EX (Thermo Fisher, Uppsala, Sweden)

### Chitin

Crab shell chitin was purchased from Sigma-Aldrich, Stockholm, Sweden. Chitin molecules are insoluble in PBS. To assure that chitin preparations were not contaminated with soluble compounds that may interfere with activation of cells, chitin supernatants were prepared as described for setae (see above) and used in control cultures.

### Preparation of peripheral blood mononuclear cells (PBMC)

Heparinized venous blood samples or buffy coats from donors were diluted two fold in PBS pH 7.4 without Ca2^+^ and Mg2^+^. The solution was poured carefully on the top on an equal volume of Ficoll Paque Plus (GE Healthcare, Uppsala, Sweden) followed by centrifugation at 450 g for 20 minutes. The interface containing the PBMC was harvested and washed twice in PBS (same as above) and centrifuged at 300 g for 10 minutes. After the last centrifugation step the cells were counted in a hemocytometer and re-suspended in RPMI tissue culture medium supplemented with L-glutamin (2 mM), penicillin (100 U/ml), streptomycin (100 µg/ml) and 5% pooled and heat-inactivated human AB^+^ serum (complete medium). The final suspension contained normally more than 80% small lymphocytes when purified from buffy coats and 90–95% when purified from venous blood samples.

### Preparation of T-lymphocytes

The T-cells were isolated from PBMC using the Pan T-cell isolation kit (Cat. no. 130-096-535) according to the manufactures instructions (Miltenyi Biotech, Bergisch Gladbach, Germany). The method is based on depletion of non-target cells (negative selection). The non-target cells were labelled by using a cocktail of biotin-conjugated monoclonal antibodies to surface markers not present on T-cells followed by binding of anti-biotin monoclonal antibody conjugated magnetic micro beads. The magnetic labelled non-target cells were depleted by retaining them on a MACS (manual cell separation) column in the magnetic field of a MACS separator while the unlabelled T-cells pass through the column. The antibody cocktail contained antibodies against CD14, CD15, CD16, CD19, CD34, CD36, CD56, CD123 and CD235a (Glycophorin A). The content of CD3+ cells after isolation was regularly>97% as analyzed by flow cytometry.

### Flow cytometry

Staining of cell surface molecules was performed according to standard procedures. In brief, cells were labelled with monoclonal specific antibodies (Ab) or the corresponding isotype control Ab conjugated with fluorescein isothiocyanate (FITC), phycoerythrin (PE) or allophycocyanin (APC). The following antibodies were used: APC mouse anti human CD3 (BD Pharmingen, Stockholm, Sweden) PE mouse anti-human CD19 and FITC mouse anti-human CD25 (Becton Dickinson, Stockholm, Sweden). The cells (0.5×10^6^ cells/tube) were washed once in ice cold PBS, pH7.4, supplemented with 0.5% bovine serum albumin (wash buffer) and centrifuged at 300 g for 5 minutes. The supernatant was discarded and the 50 µl diluted antibody was added and incubated on ice for 30 minutes. The FITC and APC conjugated antibodies were diluted 1∶5 and the PE conjugated antibodies 1∶10 in wash buffer. After incubation the cells were washed twice as above. Finally, the cells were re-suspended and fixed in 1% paraformaldehyde (PFA), pH7.4. Flow cytometry was performed on a Gallios Flow cytometer and analyzed with Kaluza software version 1.2 (Beckman Coulter, Inc. Fullerton, CA, USA).

### In vitro cell culture experiments

PBMC or enriched T-cells were suspended in RPMI tissue culture medium with L-glutamine (2 mM), penicillin (100 U/ml), streptomycin (100 µg/ml) and 5% pooled and heat-inactivated human AB serum (complete medium). In total, 0.15×10^6^ cells in 100 µl complete medium were added to each well of tissue culture plates (96-well plate, Techno Plastic Products AG, TPP, Trasadingen Switzerland). Before the cells were added to the 96-well plate, 50 µl of reactants, medium, 400 setae, setae extract (100 ng/ml) or chitin (5 µg/ml) were added to the wells. Each reactant was added to the 96-well plate in sextuplicates. The plates were incubated in a humid atmosphere with 5% CO_2_ at 37°C for 6 days in total. Eighteen hours before harvesting the cells, l µCi ^3^H-thymidine (20 Ci/mmol, GE Healthcare) was added to each well. The cell pellets were harvested and washed in a Harvester 96-Automated TOMTEC and the radioactivity was counted in a Wallac 1450 Beta scintillation counter (Perkin Elmer). The mean counts/min (ct/min) of six wells was calculated.

In parallel, cells incubated with setae, setae extract or medium only with no addition of ^3^H-thymidine were stained for CD3, CD25, and CD 19 after 6 days.

### Microscopy analysis

The interaction between setae and PBMC in cell cultures was observed by phase contrast microscopy. In order to visualize the interaction further, cytospin preparations followed by May-Grunwald Giemsa staining was performed on PBMCs after 24, 72 hours and 6 days of incubation with setae.

PBMCs were prepared from buffy coat as described above and 0.15×10^6^ cells in 100 µl complete medium were added to each well of a 96-well plate. Before the cells were added to the 96-well plate 50 µl complete medium only or medium containing 400 setae was added to each well. All the cells in one well were harvested after 24, 72 hours and 6 days. The cells were washed twice in PBS containing 5% FBS and dispersed to an object glass by cytospin centrifugation according to the protocol for cytospin 2 from Shandon (Thermo Scientific). The cells were fixed in methanol for 5 minutes and stained with May-Grunwald Giemsa according to standard procedure.

### Statistical analysis

The samples comprised 10 individuals, 4 non-exposed and 6 exposed to setae. Three of the 4 non-exposed individuals were observed repeatedly over time at visits at least 6 months apart. DNA synthesis (ct/min) was measured under four different exposures labelled medium, setae, setae extract, and chitin. Overall we analysed 55 valid observations. We utilized multilevel, hierarchical linear models to estimate the mean over combinations of sensitization and exposure. Level 1 was the measure, level 2 the time point, and level 3 the individual. Multilevel models appropriately account for the dependence within each level. Estimates and confidence intervals were obtained via maximum likelihood estimation.

## Results

### Microscopy of cultures

**Figure 1:**
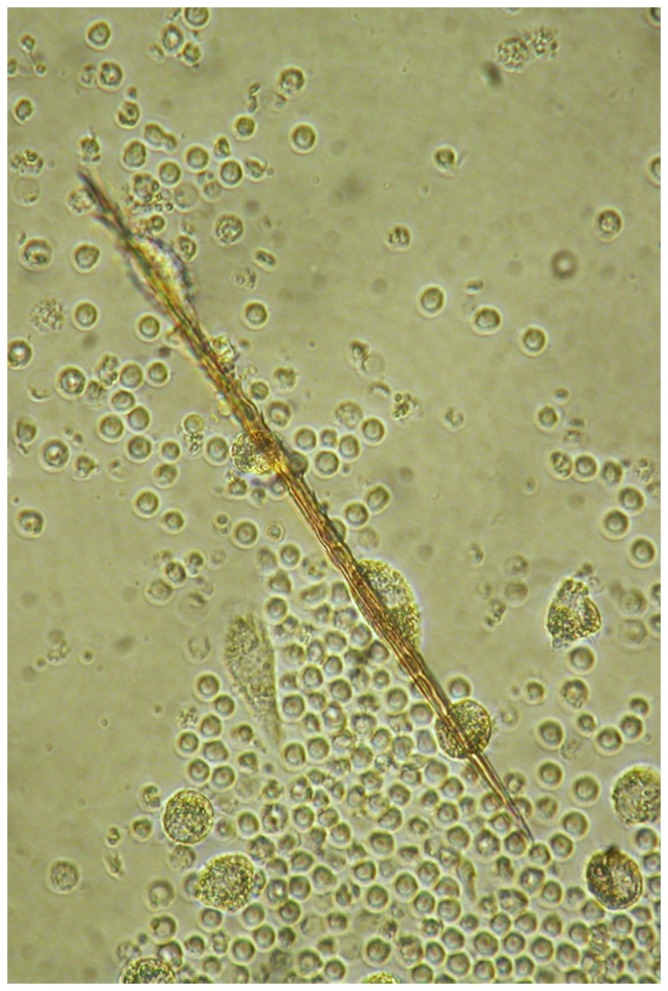
Phase contrast image of a seta in a PBMC culture from buffy coat (a non-exposed donor) after 24 hours of incubation. Mononuclear cells adhere along the setae.

**Figure 2:**
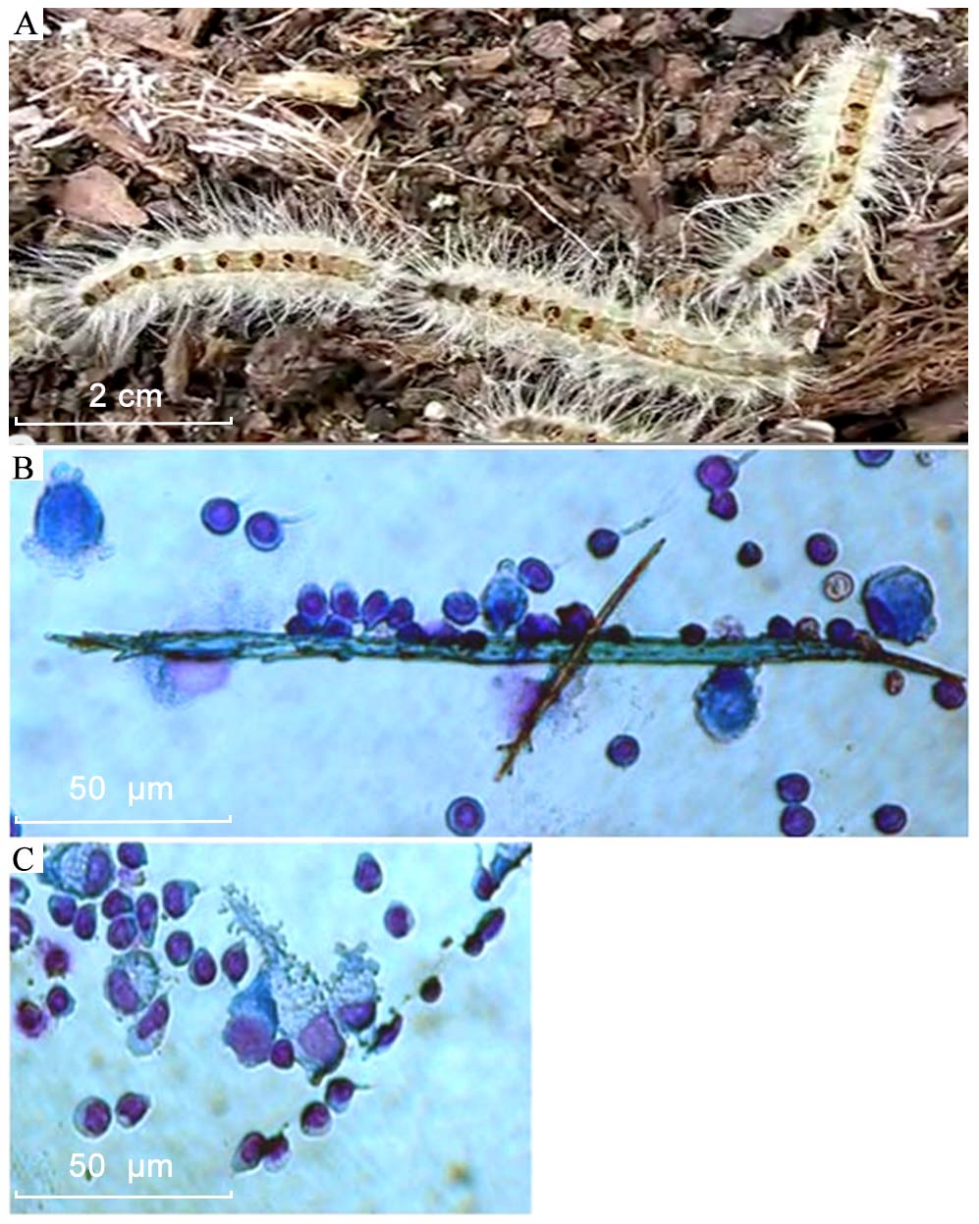
Setae from Thaumetopoea pinivora larvae and their interaction with PBMC. A. A larva resting on the ground. Note the dark, round areas (called mirrors) on the back containing the setae; B and C. Cytospin preparations of lymphoid cells cultured with setae for 24 (B) and 72 (C) hours, respectively, stained with May-Grunwald Giemsa showing adhesion of PBMC to setae and degradation of setae.

**Figure 3:**
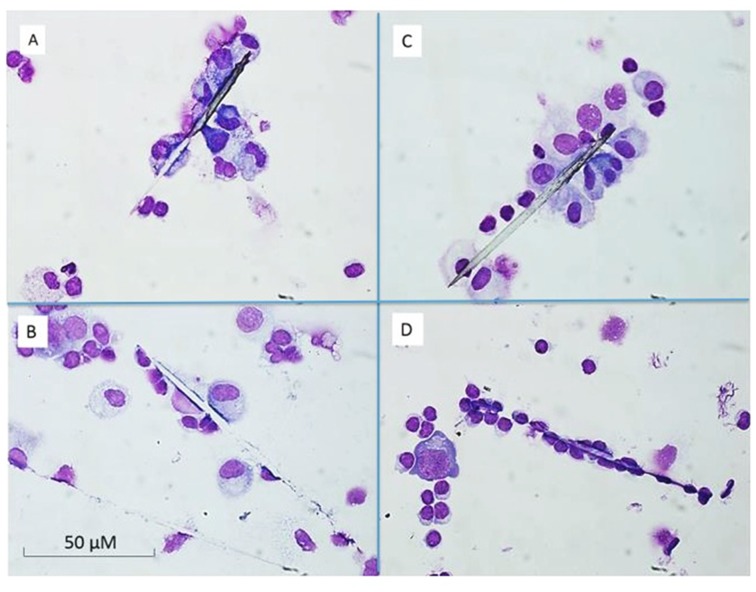
Cytospin preparations of PBMC after 6 days of incubation. A and B represents a donor earlier exposed to setae, C and D illustrates not earlier exposed donors. The microscopic view indicated no difference between exposed and non-exposed donors. In A and C mononuclear are seen cells attached to the setae B and D the shows patterns of disintegrated seta. The cultured PBMC and setae were collected by cytospin and stained with May-Grunwald Giemsa.

Using phase contrast microscopy mononuclear cells were seen to adhere to setae within 24 hours of culture and some cells are stretched along the setae (Fig. 1) that had been prepared from setae mirrors of the larvae (Fig. 2A). No damage of setae was noted at this time of incubation. As observed in stained cytospin preparations, some of the cells seemed to adhere to setae via cellular protrusions. After 72 hours of culture, large cells with pale and large nuclei and irregular cytoplasms with protrusions were seen in close contact with setae (Fig. 2C). Such setae often showed signs of disintegration (Fig. 2C). Fig. 3 illustrates the interaction between setae and PBMC from a donor earlier exposed for setae (A and B) as well as from donors never exposed to setae (C and D) after 6 days of incubation. The microscopy images showed a similar pattern of adhesion and setae damage independent of whether the donors had been exposed to setae or not (Fig. 3). In summary, mononuclear cells adhere to setae within 24 hours of incubation. Damage of seta was first observed after 72 hours of culture and proceed to disintegration of the majority of setae after 6 days.

### Cell proliferation

Setae stimulated proliferation of PBMC in cell culture as measured by ^3^H-thymidine incorporation during the last 18 hours of incubation. Longer time than 6 days of incubation may result in increased cell death, therefore the majority of the experiments was terminated after 6 days of culture. Variation of the response is determined by several factors. The size of setae varies not only between batches but also within the batches. The number of setae was routinely determined by counting under a microscope.

The stimulatory response was dose dependent with an optimum of 400 setae per micro-well (Table 1). The concentration of 400 setae was selected due to the highest mean value. The difference between 200 and 800 setae was not statistically significant. The lack of significance can be explained by the low statistical power due to the small number of observations in the groups being compared.

**Table 1:** Optimum of PBMC cell proliferation was at 400 setae/micro-well.

Additions	PBMC (ct/min±SD)
**Medium**	2575±561
**Setae 200**	33010±5708
**Setae 400**	39654±5832
**Setae 800**	36808±3743

PBMC from one donor previously exposed to setae was incubated with different numbers of setae medium (0 setae), 200 setae, 400 setae and 800 setae/micro- well. Setae were suspended in PBS and sonicated before stimulation and cell proliferation was determined by ^3^H-thymidine incorporation. The experimental setup is described in material and methods. The mean ct/min of 4–5 parallel incubations ± standard deviation (SD) are given.

Saline extracts of setae strongly stimulated lymphocyte proliferation. Setae or setae extracts did not stimulate proliferation of enriched T-lymphocytes (>97%) purified by negative selection in presence of a cocktail of monoclonal antibodies to non T-cells. This indicates that setae and setae extracts did not contain or was contaminated by any unspecific T-cell mitogenic factors. In contrast, monoclonal anti-CD3 antibodies were strong T-cell stimulators (Table 2). The fraction of activated T-cells expressing the CD25 marker was increased in setae extract stimulated cultures and to a lesser degree also in enriched T-cell cultures. The percentage of CD19+ B-cells remained low (Table 3). This indicates that a T-cell proliferation occurs only when PBMCs are present in the culture.

**Table 2:** Setae and setae extracts did not stimulate enriched T-lymphocytes under conditions where anti-CD3 was a strong stimulator as measured by ^3^H-thymidine incorporation.

Additions	PBMC (ct/min±SD)	T-cells (ct/min±SD)
**Setae 400**	19965±4253 (p<0.001)	101±56 (n.s.)
**Setae extract**	14878±6015 (p<0.001)	113±39 (n.s.)
**Medium**	394±57	84±46
**α-CD3**	200031±16421 (p<0.001)	331691±10765 (p<0.001)

The mean ct/min of six parallel incubations ± standard deviation (SD) are given. The p-values are based on comparison with medium control. PBMC and T-cells were prepared from buffy coat.

**Table 3:** Setae and setae extracts stimulate the expression of CD3 and CD25.

	CD3^+^(%)	CD3^+^CD25^+^(%)	CD19^+^(%)
**PBMC**			
Setae extract	64.2	19.5	2.5
Medium	54.0	6.4	2.1
Chitin supernatant	53.9	8.6	2.3
**T-cells**			
Setae extract	94.0	10.4	n.d.
Medium	98.4	3.0	n.d.
Chitin supernatant	98.4	2.8	n.d.

FACS analysis on CD markers for PBMC and T-cell preparations stimulated with setae extract and or chitin supernatant. PBMC and T-cells were prepared from the same buffy coat as used in the experiment depicted in Table 1.

**Figure 4:**
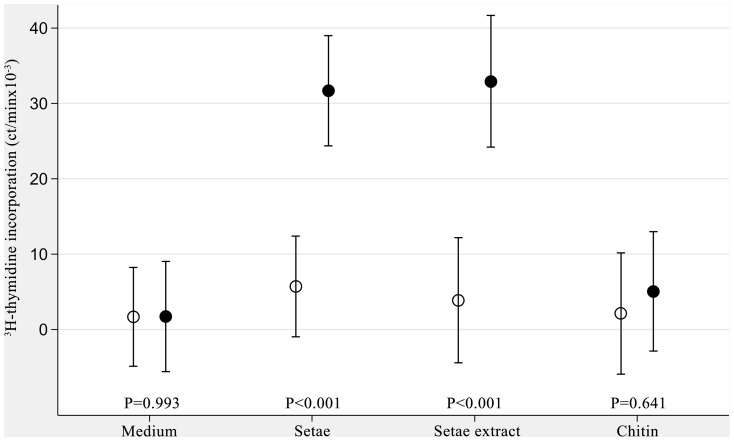
Setae and setae extracts stimulated proliferation of PBMC from setae exposed persons. Here a multilevel model estimates for mean DNA synthesis by exposure in four non-exposed (hollow dots) and six exposed (solid dots) individuals. The vertical bars indicate the 95% confidence intervals.

Persons who had been exposed to setae by living in contaminated areas displayed higher mean lymphocyte proliferation induced by setae or setae extracts than non- exposed donors (Fig. 4). The differences were statistically significant (p<0.001). The mean cell stimulation induced by crab chitin did not differ between exposed and non-exposed persons. However, chitin stimulated lymphocyte proliferation to a low but statistically significant extent independently of donors previous setae exposure (Table 4). In order to exclude the presence in the insoluble chitin contaminating lymphocyte stimulating factors, supernatants of sonicated chitin were tested in that case no cell stimulation could be detected (Table 4)

**Table 4:** Chitin enhances the stimulation of DNA synthesis in PBMC.

	Mean±SE	95%Conf. interval
**None**	2.10±0.43	1.26–2.94
**Chitin**	3.67±0.51	2.67–4.66

The results are based on multilevel model estimates for the mean for DNA synthesis (ct/min) ×1000 comparing no exposure with chitin exposure (see Materials and Methods). The difference between no exposure and chitin is statistically significant (p-value  =  0.004). SE = standard error, Conf. interval = Confidence interval.

The mean lymphocyte proliferation induced by setae or by setae extracts was significantly stronger in persons who had been exposed to setae as compared with non-exposed donors (Fig. 4).

Chitin stimulated lymphocyte proliferation to a low but statistically significant extent independently of the donors previous setae exposure (Table 4).

## Discussion

The results of the present study show for the first time that setae and saline extracts from setae harvested from larvae of TP can stimulate human blood T-lymphocyte proliferation *in vitro* while there were no signs of B-lymphocyte activation. The number of T-lymphocytes carrying the T-cell activation marker CD25 was increased. No increase of CD19+ B-cells was noted. The proliferation of T-cells induced by setae or setae extracts was critically dependent on the presence of monocytes/macrophages during cultivation, suggesting that setae and setae extract do not exert T-lymphocyte mitogenic effects.

The introduction of setae into skin and mucous membranes may lead to break-down of setae by enzymes from monocytes-macrophages and other cells thus promoting the release of setae molecules [1]. The ensuing uptake and processing of seta components by antigen presenting cells may lead to presentation to antigen-reactive T-cells, thus starting T-lymphocyte proliferation and the induction of an immune response [1]. This notion is supported by the observation that stimulation of cells from previously exposed persons by setae or setae extracts was much stronger than cells from non-exposed individuals. Hence, the ability of soluble setae components to activate T-lymphocytes in the presence of monocytes/macrophages may be regarded as an in vitro counterpart of delayed hypersensitivity reactions in sensitized persons. In fact, skin exposure of volunteers to setae resulted in local skin reactions of delayed type [3], [8], while no rapid “wheel-and-flare” reactions were observed [3].

Chitin exerted weak T-cell stimulatory capacities in some donors suggesting that setae chitin may contribute to the stimulation of lymphocytes. However, while saline extracts of setae were highly stimulatory, similar extracts from chitin did not stimulate cell proliferation. These findings suggest that chitin and soluble setae molecules act via distinct T-cell activating mechanisms. The mechanism underlying the T-cell stimulatory property of chitin action is not known. Chitin is insoluble and consequently saline extracts of crab chitin did not induce lymphocyte proliferation. It is therefore assumed that chitin has to be degraded by chitinases produced by macrophages present in PBMC, before becoming capable to activate lymphocytes. This observation is in line with studies of the immunomodulatory and proinflammatory properties of chitin showing that only chitin particles can be processed by inflammatory cells [9].

Lymphocyte activating components in setae other than chitin, are only partly known. Recently, Rodriquez-Mahillo et al [10] reported the isolation and purification of a major protein allergen in setae from *T. pityocampa* that reacted with sera from persons with IgE mediated allergy towards setae antigens. Several minor protein allergens were also identified in setae. Whether similar molecules in *T. pinivora* can induce stimulation of human T-lymphocytes remains to be shown.

In conclusion, setae from TP larvae, as well as saline extracts of setae, activated proliferation *in vitro* of human blood T-lymphocytes when cultivated in the presence of monocytes. The response was particularly strong in cells from previously exposed persons. Our findings are of interest for the understanding of the local and general processes following the penetration of setae into skin and mucous membranes, thereby introducing setae materials foreign to the immune system of the host.
